# Coxsackievirus B3 infection induces glycolysis to facilitate viral replication

**DOI:** 10.3389/fmicb.2022.962766

**Published:** 2022-12-09

**Authors:** Yujie Qian, Yeyi Yang, Wenxiang Qing, Chunyun Li, Min Kong, Zhijuan Kang, Yuanbojiao Zuo, Jiping Wu, Meng Yu, Zuocheng Yang

**Affiliations:** ^1^Department of Pediatrics, The Third Xiangya Hospital, Central South University, Changsha, China; ^2^Department of Medicine, The Third Xiangya Hospital, Central South University, Changsha, China; ^3^Department of Anesthesiology, The Third Xiangya Hospital, Central South University, Changsha, China; ^4^The Key Laboratory of Model Animals and Stem Cell Biology in Hunan Province, School of Medicine, Hunan Normal University, Changsha, China

**Keywords:** coxsackievirus B3, glycolysis, hexokinase 2, muscle phosphofructokinase, pyruvate kinase M2, autophagy, glycolysis inhibitor

## Abstract

Coxsackievirus B3 (CVB3) is a leading cause of viral myocarditis, but no effective treatment strategy against CVB3 is available. Viruses lack an inherent metabolic system and thus depend on host cellular metabolism for their benefit. In this study, we observed that CVB3 enhanced glycolysis in H9c2 rat cardiomyocytes and HL-1 mouse cardiomyocytes. Therefore, three key glycolytic enzymes, namely, hexokinase 2 (HK2), muscle phosphofructokinase (PFKM), and pyruvate kinase M2 (PKM2), were measured in CVB3-infected H9c2 and HL-1 cells. Expression levels of HK2 and PFKM, but not PKM2, were increased in CVB3-infected H9c2 cells. All three key glycolytic enzymes showed elevated expression in CVB3-infected HL-1 cells. To further investigate this, we used 2 deoxyglucose, sodium citrate, and shikonin as glycolysis inhibitors for HK2, PFKM, and PKM2, respectively. Glycolysis inhibitors significantly reduced CVB3 replication, while the glycolysis enhancer dramatically promoted it. In addition, glycolysis inhibitors decreased autophagy and accelerated autophagosome degradation. The autophagy inducer eliminated partial inhibition effects of glycolysis inhibitors on CVB3 replication. These results demonstrate that CVB3 infection enhances glycolysis and thus benefits viral replication.

## Introduction

CVB3 belongs to the virus family *Picornaviridae* and genus *Enterovirus*, and is a positive single-stranded RNA virus, which is a leading causative agent of viral myocarditis (VMC) (Rivadeneyra et al., 2018). VMC is myocardial inflammation due to a virus infection that can lead to severe heart muscle injury, resulting in dilated cardiomyopathy and sudden cardiac death (Lee et al., 2017). CVB3 infection begins by coupling of the virus to host cell receptors, and no treatment is effective in preventing CVB3 infection at present (Selinka et al., 2004).

Viruses rely on host cell factors to replicate, modifying cellular metabolism to complete their life cycle. Most viruses examined to date induce aerobic glycolysis, also known as the Warburg effect, which means glucose is primarily utilized to produce lactic acid even in the presence of abundant oxygen. Glycolysis favors virus infections as it produces rapid energy (Pfeiffer et al., 2001; Chi et al., 2018), generates precursors (Vastag et al., 2011; Fernandes-Siqueira et al., 2018; Gualdoni et al., 2018), and restores the host redox balance (Chen et al., 2016). While oxidative phosphorylation provides significantly more ATP per glucose molecule, glycolysis is a much faster process that rapidly provides ATP. The virus also activates glycolysis to generate precursors for nucleotide and protein synthesis required for viral replication. Glycolysis simultaneously elevates the NADH/NAD^+^ ratio to counteract the high levels of reactive oxygen species produced in response to virus infection. Thus, most viruses require glycolysis for optimal replication. But, some viruses, such as vaccinia virus, do not induce glycolysis for replication (Fontaine et al., 2014).

Despite extensive explorations in virus infection and glycolysis (Polcicova et al., 2020), little is known about glucose metabolomic change in CVB3-infected cardiomyocytes. Therefore, we propose a hypothesis regarding glucose metabolism changes in CVB3-infected cardiomyocytes. We also present relevant experiments using myocardial cell lines, namely, H9c2 rat cardiomyocytes and HL-1 mouse cardiomyocytes, to support this hypothesis. The present study reveals that glucose metabolism is involved in CVB3 infection and is required for CVB3 replication.

## Materials and methods

### Cell culture

The myocardial cell lines H9c2 and HL-1 were obtained from the Institute of Oncology, Central South University, China, and grown in Dulbecco's modified Eagle medium (DMEM, Gibco, Life Technologies, Inc, Waltham, MA, USA) supplemented with 10% heat-inactivated fetal bovine serum (FBS, Gibco), and Claycomb medium (Sigma-Aldrich, St. Louis, MO USA) with 10% FBS, respectively. The cells were cultured at 37°C in a humidified incubator with 5% CO_2_.

### Virus infection

Coxsackievirus B3 (Nancy strain) was obtained from Shanghai Jiao Tong University School of Medicine and propagated and maintained in H9c2 cells. The viral titer was routinely examined prior to each experiment. The cells were washed with phosphate-buffered saline (PBS), serum-starved for 2 h prior to infection, and then mock-infected or CVB3-infected at a multiplicity of infection of 10 in serum-free medium for 1 h.

### Glycolytic inhibitor treatment and stimulation experiment

The cells were infected with CVB3 at a multiplicity of infection of 10 in a serum-free medium for 1 h. 2-Deoxy-d-glucose (2DG) (Sigma-Aldrich, Germany) and sodium citrate (SCT) (Sigma-Aldrich, Germany) were solubilized in cell culture medium for each experiment and added to the experimental medium at a final concentration of 10 mM. Shikonin (Sigma-Aldrich, Germany) was solubilized in DMSO for each experiment and added to the experimental medium at a final concentration of 1 μM. For glycolysis inhibitor studies, CVB3-infected cells were treated with glycolysis inhibitors, namely, 2DG, SCT, and shikonin, at indicated concentrations. PS48 (Abcam, Cambridge, UK) and rapamycin (Sigma-Aldrich, Germany) were solubilized in DMSO for each experiment and added to the experimental medium at indicated concentrations. For glycolysis activation experiments, CVB3-infected cells were treated with 10 mM PS48. For autophagy induction experiments, CVB3-infected cells were treated with 100 nM rapamycin.

### Plaque assay

The virus titer in the cell supernatant was determined using an agar overlay plaque assay, as previously described (Si et al., 2007). In brief, the cells were infected with serial 10-fold dilutions of the virus supernatants for 1 h. Then, the cells were washed with PBS and covered with a medium containing 0.75% agarose. After 72 h of incubation, the plates were stained with 1% crystal violet The visible plaques were counted, and the viral titer was calculated as plaque-forming units per milliliter.

### Glucose consumption and lactate production

Glucose consumption and lactate production were measured by using a glucose assay kit (BC2500, Solarbio, Beijing, China) and a lactate assay kit (BC2230, Solarbio, China) in accordance with the manufacturers' instructions. All data were normalized to the cell numbers.

### Cell counting kit-8 assay

To evaluate the chemical cytotoxicity, cell counting kit-8 (CCK-8) experiments were performed following the manufacturer's instructions. In brief, the cells were seeded in 96-well plates at a density of 5 × 10^3^ cells/ml for 24 h and then exposed to the drugs or controls for 0, 6, 12, and 24 h. The cells were analyzed by adding 5 mg/ml of the CCK-8 reagent (Vazyme Biotech, Nanjing, China). After a 2-h incubation, absorbance was measured by wavelength spectrophotometry at 450 nm.

### Creatine kinase isoenzyme MB measurements

The cells were seeded in six-well plates for 24 h at 6 × 10^5^ cells per well and then exposed to the drugs or controls. The medium was collected at each time point, and the creatine kinase-MB (CK-MB) levels were immediately examined using an Olympus (Tokyo, Japan) AU5400 analyzer.

### Real-time quantitative polymerase chain reaction assay

The TaqMan software was used to design the real-time quantitative polymerase chain reaction (RT-qPCR) based on exon junctions to prevent co-amplification of genomic DNA. Total mRNA was extracted using an RNA extraction kit (Omega Bio-Tek, Norcross, GA, USA) and reverse-transcribed into cDNA using a PrimeScript RT reagent kit with a gDNA eraser (Takara, Kusatsu, Japan). The primers used for the genes indicated were designed using Primer Blast. The primer sequences are listed in Tables 1, 2. The reaction was performed using a LightCycler^®^ 480II analyzer (Roche, Mannheim, Germany), and the amplifications were carried out for 35 cycles. β-Actin was used as the internal control, and the 2^−ΔCt^ method was used to calculate the differences in mRNA transcript levels.

**Table 1:** Primer sequences for H9c2 rat cardiomyocytes.

**Target gene**	**Primers**	**Sequence (5′-3′)**	**Amplicon length (bp)**
CVB3	Forward	ACGAATCCCAGTGTGTTTTGG	67
	Reverse	TGCTCAAAAACGGTATGGACAT	
GLUT4	Forward	GCCTGCCCGAAAGAGTCTAA	275
	Reverse	CAGCTCCTATGGTGGCGTAG	
HK2	Forward	AGGGATTCAAGGCATCTGGC	86
	Reverse	CAGGTCAAACTCCTCTCGCC	
PFKM	Forward	CCATCAGCCTTTGACCGGAT	165
	Reverse	GGTCACGTCTTTGGTCACCT	
PKM2	Forward	ATGAAGGTGTCCGCAGGTTT	137
	Reverse	TCGGTTGCATCGTCCAATCA	
Atg5	Forward	ACCTCGGTTTGGCTTGGTT	103
	Reverse	AAACCACACGTCTCGAAGCA	
Beclin1	Forward	CCCAGCCAGGATGATGTCTAC	96
	Reverse	AGTCTCCGGCTGAGGTTCTC	
β-Actin	Forward	CGCGAGTACAACCTTCTTGC	70
	Reverse	CGTCATCCATGGCGAACTGG	

**Table 2:** Primers sequences for HL-1 mouse cardiomyocytes.

**Target gene**	**Primers**	**Sequence (5′-3′)**	**Amplicon length (bp)**
CVB3	Forward	ACGAATCCCAGTGTGTTTTGG	67
	Reverse	TGCTCAAAAACGGTATGGACAT	
GLUT4	Forward	AAACAAGATGCCGTCGGGT	173
	Reverse	AGCTCTGTTCAATCACCTTCTG	
HK2	Forward	GGCTAGGAGCTACCACACAC	91
	Reverse	AACTCGCCATGTTCTGTCCC	
PFKM	Forward	ATTCGCGATCTCCAGGTGAA	211
	Reverse	CAAAGGGAGTTGGGCTTCCA	
PKM2	Forward	GCATGCAGCACCTGATAGCTC	78
	Reverse	AGGCTCGCACAAGCTCTTCA	
Atg5	Forward	TGCATCAAGTTCAGCTCTTCC	224
	Reverse	ACTGGTCAAATCTGTCATTCTGC	
Beclin1	Forward	CCAGCCTCTGAAACTGGACA	82
	Reverse	TGTGGTAAGTAATGGAGCTGTGA	
β-Actin	Forward	CACTGTCGAGTCGCGTCCA	88
	Reverse	CATCCATGGCGAACTGGTGG	

### Western blot analysis

The cells were washed with PBS and then lysed on ice in a radioimmunoprecipitation assay (RIPA) buffer (Kangwei, Beijing, China). The total protein concentration was measured by bicinchoninic acid (BCA) protein assay (CWBIO, Beijing, China). Subsequently, 30 μg of protein was subjected to SDS-PAGE and subsequently transferred to PVDF membranes (Millipore Corporation, Billerica, MA, United States). The membranes were blocked for 1 h with 5% non-fat milk before being incubated with primary antibodies [HK2, PKM2, GLUT4: Cell Signaling Technology, Danvers, USA. LC3-I/II: Sigma-Aldrich, Germany; ATG5, Beclin1, PFKM, β-actin: Proteintech Group, Inc., Chicago, IL, USA; monoclonal anti-enterovirus antibody (viral capsid protein, VP1) Dako, Carpinteria, CA, USA] at 4°C overnight, followed by incubation with an IRDye^®^800CW goat anti-rabbit secondary antibody (1:8,000, 926-32211, LI-COR^®^, USA) for another 1 h. Immunoblotting bands were visualized under an Odyssey CLx infrared imaging system (LI-COR^®^, USA). Protein expression levels were quantified by densitometry using the ImageJ software and were normalized to β-actin.

### Statistical analysis

Data were presented as the mean ± standard error of the mean (SEM), and statistical analyses were performed by using SPSS software version 18.0. Student's *t*-test was used to determine statistical significance. Results with a *P* < 0.05 were considered statistically significant.

## Results

### CVB3 infection induces glycolysis

**Figure 1:**
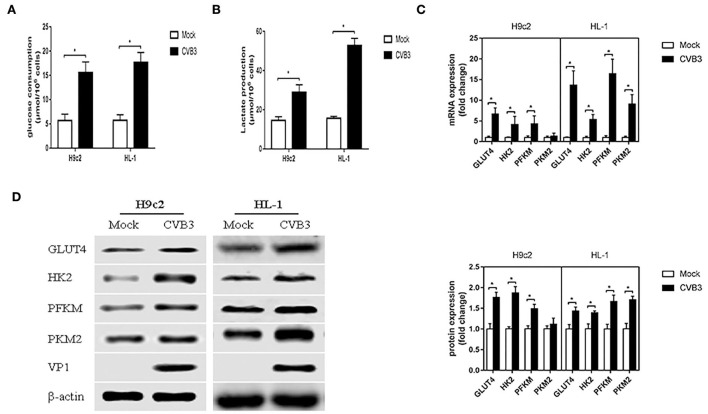
Coxsackievirus B3 infection induces glycolysis. Myocardial cells, H9c2 and HL-1 cells, were mock-infected or infected with coxsackievirus B3 at a multiplicity of infection of 10, and were harvested at 24 h post-infection. Glucose consumption **(A)** and lactate production **(B)** were measured and calculated. The expressions of GLUT4, HK2, PFKM, and PKM2 were analyzed by qPCR **(C)** and Western blot **(D)**. Data are indicated as mean ± SEM; data represent three independent experiments with three technical replicates per experiment; *P < 0.05.

To determine whether CVB3 induces glycolysis in myocardial cells, H9c2 and HL-1 cells were infected with CVB3 to establish cellular models of viral myocarditis (Tabor-Godwin et al., 2012; Li et al., 2014, 2019; Wang et al., 2018; Li and Xie, 2022; Pappritz et al., 2022). We measured glucose consumption and lactate production, which approximatelyreflect fluctuations in glycolysis (Sun et al., 2011; Mazzon et al., 2018; Lee et al., 2020). Compared with the mock group, the CVB3 group showed an increase in glucose consumption (*P* < 0.05; Figure 1A). Meanwhile, the lactate level in the CVB3 group was higher than that in the mock group (*P* < 0.05; Figure 1B). The increase in glucose consumption and lactate production suggests a possible increase in glucose transport and metabolism. To confirm this speculation, we detected the expression of glucose transporter 4 (GLUT4), which is mainly expressed in the myocardial cells and transported glucose into cells (Wang et al., 2020). The key glycolytic enzymes hexokinase 2 (HK2), muscle phosphofructokinase (PFKM), and pyruvate kinase M2 (PKM2) were detected. Indeed, compared with the mock group, the expression of GLUT4 in CVB3-infected myocardial cells was increased. Expression levels of HK2 and PFKM, but not PKM2, were increased in CVB3-infected H9c2 cells. All three key glycolytic enzymes showed elevated expression in CVB3-infected HL-1 cells (Figures 1C,D). Taken together, these results suggest that glycolysis was enhanced after CVB3 infection in H9c2 and HL-1 cells.

### Glycolysis inhibitors reduce CVB3 replication

**Figure 2:**
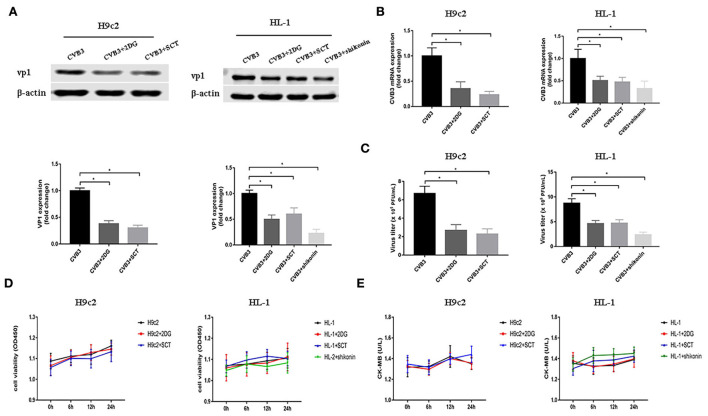
Glycolysis inhibitors reduce CVB3 replication. H9c2 and HL-1 cells were infected with coxsackievirus B3 (CVB3) at a multiplicity of infection of 10, with or without glycolytic inhibitor treatment, and were harvested at 24 h post-infection. Intracellular VP1 levels were measured by Western blotting **(A)**, and CVB3 RNA levels were measured by quantitative PCR **(B)**. Cell supernatant was harvested at 24 h post-infection, and CVB3 viral titer levels were measured by plaque forming unit assay **(C)**. Myocardial cells treated with or without 2-deoxy-D-glucose, sodium citrate, or shikonin were detected at 0, 6, 12, and 24 h by using the CCK-8 method **(D)**, and the culture supernatant creatine kinase MB levels were tested at the same time points **(E)**. Data are indicated as mean ± SEM; data represent three independent experiments with at least three technical replicates per experiment; *P < 0.05.

As demonstrated before, glycolysis was elevated after CVB3 infection. Previous studies have shown that metabolic reprogramming is beneficial for viral replication in host cells (Thai et al., 2014, 2015; Fontaine et al., 2015; Passalacqua et al., 2019). To examine whether the disruption of glycolysis affects the replication of CVB3, we utilized 2DG and SCT as glycolysis inhibitors to inhibit the increased HK2 and PFKM expression levels in H9c2 cells, respectively, and 2DG, SCT, and shikonin as glycolysis inhibitors to inhibit the increased HK2, PFKM, and PKM2 expression levels in HL-1 cells, respectively. Intracellular viral capsid protein VP1 and CVB3 RNA were detected by Western blotting and qPCR, respectively. As shown in Figures 2A,B, exposure to glycolysis inhibitors dramatically decreased CVB3 replication. Furthermore, the CVB3 titer in the cell supernatant was also significantly reduced upon glycolysis inhibitor treatment (Figure 2C). To exclude drug cytotoxicity as the mediator of inhibition, viability assays were undertaken with each compound. Uninfected cells were incubated with 2DG, SCT, or shikonin. The inhibitors did not elicit measurable effects on cell viability at any time points, and the culture supernatant did not show an increased level of CK-MB relative to the untreated controls (Figures 2D,E). These exclude the possibility that glycolysis inhibitor toxicity may impair myocardial cells.

### Enhancing the glycolysis pathway promotes CVB3 replication

**Figure 3:**
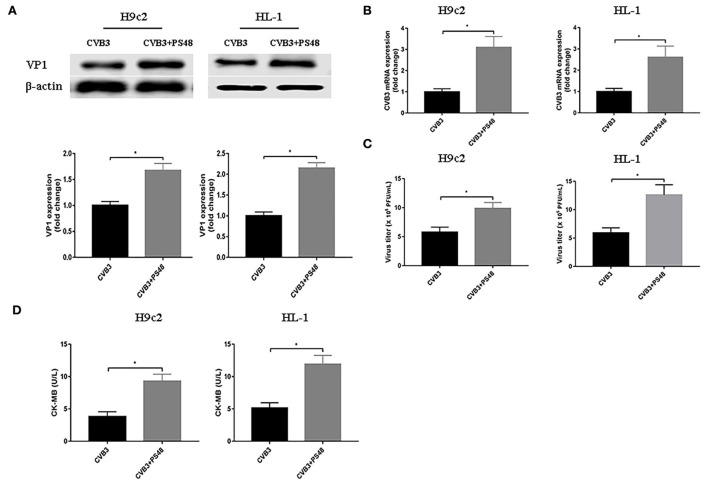
Glycolysis enhancer promotes coxsackievirus B3 replication. H9c2 and HL-1 cells were infected with coxsackievirus B3 at a multiplicity of infection of 10, with or without simultaneous PS48 (10 mM) treatment. Cells were harvested at 24 h post-infection, and the intracellular VP1 level was measured by Western blotting **(A)**, and viral RNA was measured by quantitative PCR **(B)**. Cell supernatants were harvested at 24 h post-infection, viral titer levels were measured by plaque-forming unit assay **(C)**, and creatine kinase-MB levels were tested **(D)**. Data are indicated as mean ± SEM; Data represent three independent experiments, with three technical replicates per experiment; *P < 0.05.

To further confirm the effect of enhanced glycolysis on CVB3 replication, we used PS48 to induce a Warburg-like metabolic state. PS48 shifts glucose metabolism from the TCA cycle to glycolysis by activating pyruvate dehydrogenase kinase 1 (PDK1) (Hindie et al., 2009; Han et al., 2017). As shown in Figure 3, exposure to PS48 dramatically promoted CVB3 replication. As viral replication increased, PS48 aggravated CVB3-infected cell injury.

### Glycolysis promotes CVB3 replication *via* autophagy pathway

**Figure 4:**
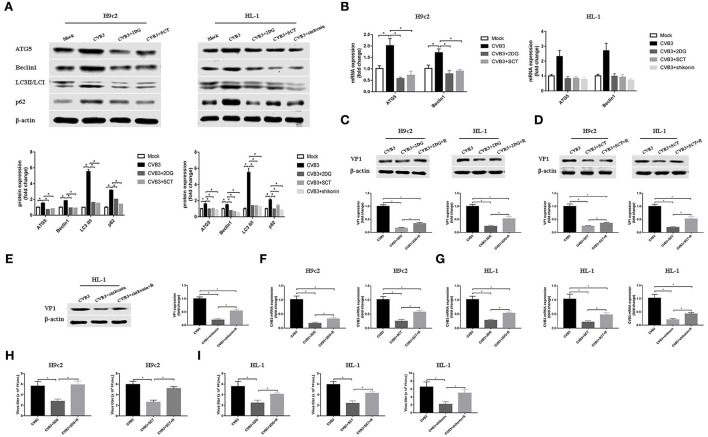
Coxsackievirus B3 replication is enhanced by activating autophagy. H9c2 cells were divided into four groups: a mock-infected group, coxsackievirus B3 (CVB3)-infected group, CVB3-infected group treated with 2-deoxy-D-glucose, and CVB3-infected group treated with sodium citrate. HL-1 cells were divided into five groups: a mock-infected group, CVB3-infected group, CVB3-infected group treated with 2-deoxy-D-glucose, CVB3-infected group treated with sodium citrate, and CVB3-infected group treated with shikonin, and cells were harvested at 24 h post-infection. ATG5, Beclin1, LC3 II,/I, and p62 were measured by Western blotting **(A)**, and ATG5 and Beclin1 were further measured using quantitative PCR **(B)**. Subsequently, cells were infected with CVB3 at a multiplicity of infection of 10 and treated with glycolysis inhibitors, with or without rapamycin (100 nM), and cells were harvested at 24 h post-infection. VP1 levels were measured by Western blotting **(C–E)**, and CVB3 RNA levels were measured by quantitative PCR **(F,G)**. Cell supernatant was harvested at 24 h post-infection, and CVB3 viral titer levels were measured by plaque-forming unit assay **(H,I)**. R, rapamycin. Data are indicated as mean ± SEM; data represent three independent experiments with three technical replicates per experiment; *P < 0.05.

Autophagy is involved in the replication of many viruses (Wang et al., 2019; Kong et al., 2020; Zhang et al., 2021). In order to investigate how glycolysis affects CVB3 replication, we detected the key protein of the autophagy pathway. We found that CVB3 infection increased the expression of ATG5 and Beclin1, and the LC3II/LC3I ratio, which was indicative of a higher level of autophagy. Concurrently, the level of p62 increased, pointing to p62 accumulation and autophagosome degradation blockage (Figures 4A,B). The high level of autophagy and autophagosome degradation blockage served as an excellent platform for viral replication and efficiently promoted virus replication, as shown in previous studies (Mao et al., 2019; Wang et al., 2019; Lin et al., 2020; Li et al., 2021). It is therefore possible that CVB3 may utilize enhanced autophagy and blocked autophagosome degradation to benefit its replication. Furthermore, we used glycolysis inhibitors to test whether glycolysis inhibition affects CVB3-induced autophagy in H9c2 and HL-1 cells. We found that exposure to glycolysis inhibitors dramatically decreased ATG5 and Beclin1 expression levels, further accelerating the degradation of autophagosomes as the LC3II/LC3I ratio and p62 expression decreased (Figures 4A,B). These suggested that CVB3 may benefit its replication by inhibiting glycolysis and promoting autophagy. To further determine if autophagy was important for CVB3 replication, we used rapamycin as an autophagy activator. Compared with glycolysis inhibitor-treated CVB3-infected cells, rapamycin increased CVB3 replication and the yields of CVB3 viral progeny (Figure 4).

## Discussion

In this study, CVB3 infection altered glycolysis, fostering favorable intracellular conditions for virus replication *via* autophagy. Consistent with most viruses that induce glycolysis (Sanchez and Lagunoff, 2015), CVB3 infection elevated glucose consumption and GLUT4, the most abundant form of glucose transporters (GLUTs) by which glucose uptake into cardiomyocytes, in cardiomyocyte cell lines H9c2 and HL-1. Viruses can accelerate glucose uptake to match the increased metabolic rate, through the upregulation of GLUT4, a more efficient glucose transporter (Yu et al., 2011). GLUTs may increase in a viral MOI-dependent manner in virus infection (Lee et al., 2020). In addition to an increased requirement for glucose, there was an increase in lactate production, the terminal product of glycolysis, in CVB3-infected cardiomyocyte cells. Most virus-infected cells presented an increase in glucose consumption, accompanied by an increase in lactate production (El-Bacha et al., 2004; Chen et al., 2011; Ramiere et al., 2014). However, the virus can also induce a glycolytic flux through a lactate-independent pathway (Lee et al., 2020). The key glycolytic enzymes were elevated, as in the case of most of the studies reporting on glycolysis in virus infection (Ren et al., 2021). However, changes in the key glycolytic enzymes were not always consistent. As shown in white spot syndrome virus (WSSV) infection, expressions of hexokinase and phosphofructokinase were increased, but not pyruvate kinase (Ng et al., 2022). Furthermore, pyruvate kinase activity was also inhibited by WSSV infection (Chen et al., 2016). Several viral proteins have been reported to interact with pyruvate kinase as a critical factor in viral pathogenesis (Zwerschke et al., 1999; Wu et al., 2008; Wei et al., 2012). It is interesting to find some changes in PKM2 expression in CVB3-infected H9c2 cells, while HK2 and PFKM increased in CVB3-infected H9c2 cells, and all the three key glycolytic enzymes increased in CVB3-infected HL-1 cells. This may be caused by the different metabolic stress to virus infection of different cell lines. The results indicate that glycolysis is activated in CVB3 infection.

Using glycolysis key enzyme inhibitors and a glycolysis enhancer in CVB3-infected H9c2 and HL-1 cells, we found that glycolysis is indeed required for viral replication. Our data are consistent with other studies on several other virus infections (Findlay and Ulaeto, 2015; Passalacqua et al., 2019; Ren et al., 2021; Zhou et al., 2021). These data illustrate that several viruses, including CVB3, require glycolysis for optimal viral replication as it provides rapid energy, substrates, and immune escape. However, unlike most viruses examined to date, vaccinia virus does not activate glycolysis to facilitate viral replication (Fontaine et al., 2014). Furthermore, our results showed that CVB3 infection triggered autophagy but impaired an autophagy flux as massive amounts of p62 accumulated. The cells respond to various stressors, including virus infection, by triggering autophagy, thereby allowing cells to survive. CVB3 infection induces autophagy and exploits this process to gain a replication advantage (Tanaka et al., 2000; Wong et al., 2008; Shi et al., 2016; Meng et al., 2020; Mohamud et al., 2021). Although previous studies have considered that CVB3 infection activates autophagy, favoring viral replication, the relationship between glycolysis and autophagy in CVB3 replication remains unclear. In our study, glycolysis inhibitors impaired CVB3 replication by downregulating the autophagy pathway and accelerating the autophagosome degradation. An autophagy inducer eliminates, at least in part, the effect of glycolysis inhibition on CVB3 replication, indicating that the effect of glycolysis on CVB3 replication may be partly dependent on autophagy. However, future studies will be needed to elucidate this mechanism. Interestingly, in dengue virus infection, inhibition of glycolysis decreases viral replication but increases the autophagy pathway (Lee et al., 2020). This discrepancy indicates a complex and unknown regulation mechanism influencing the relationship between glycolysis and autophagy in virus infection.

In summary, we found CVB3 infection increased glucose consumption, expression levels of GLUT4 and key glycolytic enzymes, and autophagic activity in myocardial cells. As glycolysis inhibitors reduced CVB3 replication, it appears as a promising target for the treatment of viral myocarditis. The limitation of this study is that we did not set up a control group with mock infection to detect CVB3 replication in glycolysis inhibition experiments and glycolysis activation experiments. Setting up control groups with a mock group would be more rigorous.

## Data availability statement

The raw data supporting the conclusions of this article will be made available by the authors, without undue reservation.

## Author contributions

Conceptualization, funding acquisition, project administration, supervision, validation, and writing—review and editing: ZY. Data curation: WQ, MK, YZ, and MY. Formal analysis: ZK and YZ. Investigation: YQ and YY. Methodology: YQ, YY, WQ, and MK. Software: WQ, ZK, and JW. Writing—original draft: YQ. All authors contributed to the article and approved the submitted version.
